# Chapter 9 Survey on Big Data Applications

**DOI:** 10.1007/978-3-030-53199-7_9

**Published:** 2020-06-20

**Authors:** Valentina Janev, Dea Pujić, Marko Jelić, Maria-Esther Vidal

**Affiliations:** 8 Institute Mihajlo Pupin, University of Belgrade, Belgrade, Serbia; 9 ADAPT SFI Centre, O’Reilly Institute, Trinity College Dublin, Dublin, Ireland; 10 CEPLAS, Botanical Institute, University of Cologne, Cologne, Germany; 11 Institute of Logic and Computation, Faculty of Informatics, TU Wien, Wien, Austria; 12 Institute Mihajlo Pupin, University of Belgrade, Belgrade, Serbia; 13 TIB Leibniz Information Centre For Science and Technology, Hannover, Germany

## Abstract

The goal of this chapter is to shed light on different types of big data applications needed in various industries including healthcare, transportation, energy, banking and insurance, digital media and e-commerce, environment, safety and security, telecommunications, and manufacturing. In response to the problems of analyzing large-scale data, different tools, techniques, and technologies have bee developed and are available for experimentation. In our analysis, we focused on literature (review articles) accessible via the Elsevier ScienceDirect service and the Springer Link service from more recent years, mainly from the last two decades. For the selected industries, this chapter also discusses challenges that can be addressed and overcome using the semantic processing approaches and knowledge reasoning approaches discussed in this book.

## Introduction

In the last decade, the **Big Data** paradigm has gain momentum and is generally employed by businesses on a large scale to create value that surpasses the investment and maintenance costs of data. Novel applications have been created for different industries allowing (1) storing as much data as possible in a cost-effective manner (volume-based value); (2) rapid analysis capabilities (velocity-based value); (3) structured and unstructured data to be harvested, stored, and used simultaneously (variety-based value); (4) accuracy of data processing (Veracity-based value); etc. In the next decade, the amount of data will continue to grow and is expected to reach 175 zetabytes in 2025 [85]. This will fundamentally affect worldwide enterprises. This chapter is interested in identifying:

- **RQ1**: What are the main application areas of big data analytics and the specific data processing aspects that drive value for a selected industry domain?
- **RQ2**: Which are the main tools, techniques, and technologies available for experimentation in the field of big data analytics?

**Fig. 1.**
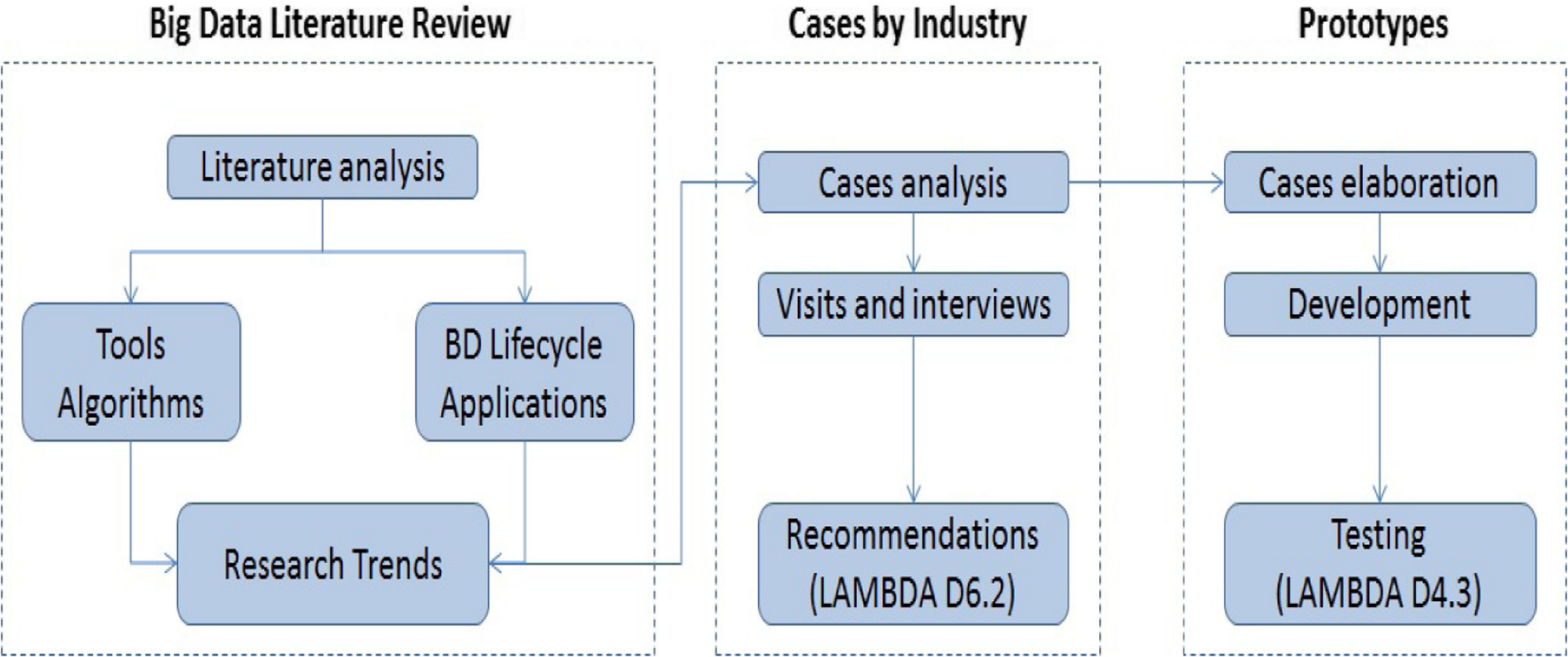
Research methodology

In December 2018, within the LAMBDA project framework, a literature review was initiated that included an extensive and comprehensive analysis of journal articles from available sources such as (1) the Elsevier ScienceDirect service^1^ and (2) the Springer Link service^2^. Elsevier ScienceDirect is a website which provides subscription-based access to a large database of scientific and medical research. It hosts over 12 million pieces of content from 3,500 academic journals and 34,000 e-books. SpringerLink is the world’s most comprehensive online collection of scientific, technological and medical journals, books and reference works printed from Springer-Verlag. In parallel, the market of available commercial and open-source tools was surveyed and monitored^3^. As **Big Data** is a very active area of research nowadays, we are also involved in analysis of different industry cases studies, as is presented in the research methodology depicted in Fig. 1. This chapter outlines the methodology and the process of selecting articles relevant for our research (see Sect. 2) and discusses the main research trends in big data applications in different industries (Sect. 3). In order to answer the second research question, the authors established the catalog of big data tools that is available at the LAMBDA project web page^4^.

## Literature Review

This section presents the literature review approach that was adopted in order to identify the relevant application areas of big data technologies. In April 2020, a simple keyword based query on term *Big Data Analytics* returns:

- 180,736 results in ScienceDirect (or 3% more than in December 2019, 174,470 results), 10,042 of them review articles, where the oldest 2 papers are from 1989 and discuss the challenges to computational science and use of supercomputers for conducting experiments in key scientific and engineering areas such as atmospheric science, astronomy, materials science, molecular biology, aerodynamics, and elementary particle physics [467];
- 40,317 results in SpringerLink (or 7% more than in December 2019, 33,249 results), where the oldest publications dating from 1950s are related to mathematics.

**Big Data Analytics** is a broad topic that, depending on the objectives of the research, can be linked on the one hand to data science and machine learning, and on the other to data and software engineering. Being interested in the role that analytics plays in business strategy, we limited our search to articles in the domain of business intelligence. Business intelligence entails the analysis of past and present data to create actionable insights for informed decision-making. Thus, the search for *review articles* linked to **Big Data Analytics** and **Business Intelligence** leads to 615 articles. The number is even smaller if we are looking for **Business Intelligence** (BI) and **NoSQL** solutions– see Table 1. That means that the concept of **Business Intelligence** still prevails in the scientific literature but is based on relational database-driven applications. Further on, looking for the year of publication, the authors have found that there are articles from the 1930s also linked to the topic **Big Data** albeit mainly related to medical studies. In our analysis, we focused on review articles from more recent years, mainly from the last two decades.

**Table 1.** Number of review articles in ScienceDirect database

Keywords	1995–1999	2000–2005	2006–2009	2010–2015	2016–2020	Total
BDA	388	718	1349	2190	4,605	10,042
BDA and BI	12	15	45	80	437	615
BDA and BI and NoSQL				3	31	35
BDA and Apps and NoSQL				8	46	54

Hence, in order to identify the main application area, we first identified journals (using ScienceDirect service) that most frequently publish research articles about **Big Data Analytics**, **Business Intelligence**, and **Applications** in **Industry**. ThetTable below points to a number of articles published in international journals between 2015 and 2019, as well as the journals relevant for the search criteria. What can be noticed is that there are three times more articles related to **Big Data and Applications**, then to **Big Data Analytics and Applications**. The number of retrieved results is drastically smaller if we introduce the topic ‘**Business Intelligence**’.

**Table 2.** Journals that match the search criteria

**‘Big Data’ and ‘Application’ (128,033)**	Neurocomputing, Journal of Cleaner Production, Procedia Computer Science, IFAC Proceedings Volumes, Expert Systems with Applications, Physica A: Statistical Mechanics and its Applications, Sensors and Actuators B: Chemical, Journal of Chromatography A, Nuclear Physics B, European Journal of Operational Research
**‘Big Data’ and ‘Industry’ (59,734)**	Journal of Cleaner Production, Future Generation Computer Systems, Energy Policy, Journal of Membrane Science, Expert Systems with Applications, Procedia Computer Science, Journal of Banking and Finance, Research Policy, European Journal of Operational Research
**‘Big Data Analytics’ and ‘Applications’ (41,031)**	Journal of Cleaner Production, Future Generation Computer Systems, Neurocomputing, Journal of Chromatography A, IFAC Proceedings Volumes, Physica A: Statistical Mechanics and its Applications, Sensors and Actuators B: Chemical, Analytica Chimica Acta, Journal of Membrane Science, Nuclear Physics B
**‘Big Data Analytics’ and ‘Business Intelligence’ (3,539)**	Future Generation Computer Systems, Procedia Computer Science, Technological Forecasting and Social Change, Expert Systems with Applications, Decision Support Systems, IFAC Proceedings Volumes, Accounting, Organizations and Society

Some of the journals listed in Table 2 refer to scientific fields that are not in direct relation to the research conducted in the LAMBDA project, such as Nuclear Physics and Astrophysics, Materials Science, Construction and Architecture, Chemistry and Chromatography. Big data research is conducted in these disciplines and there is a need for enhanced statistical algorithms, modeling and simulation approaches; however, these scientific areas are currently beyond the scope of our research and will not be discussed in the following sections.

**Trends:** Detailed analysis of the retrieved surveys on **BDA and Apps and NoSQL** (54 papers) showed that there is a shift of focus from operational data management systems, data-warehouses and business intelligent solutions (present for instance in Finance and Insurance domain in 1990s) [336] to parallel and distributed computing [478], as well as scalable architectures [187] for storing and processing data in the cloud (“Analytics in Cloud” [368]). Emerging paradigms such as the Internet of Things [120, 369] and blockchain additionally influence cloud computing systems [157]. Interconnected technologies like RFID (Radio Frequency IDentification) and WSAN (Wireless Sensor and Actor Networks) enabled development of smart environments [122] that will be explored further in subsequent sections. Wide availability of cheap processing power and vast amounts of data in recent years have enabled impressive breakthroughs in machine learning [123, 178, 269], semantic computing [222, 316], artificial neural networks and multimodal affective analytics [400].

## Big Data Analytics in Industrial Sectors

The analysis presented in this section examines the BDA-driven applications in sectors spanning healthcare, transport, telecommunications, energy production and smart grids, energy consumption and home automation, finance, media, e-Government [220] and other public utilities. The research was motivated by the needs of the Mihajlo Pupin Institute to innovate the existing product portfolio that is currently mainly focused on building advanced analytical services for control, monitoring and management of large facilities, for instance from the transport and the energy sector.

**Healthcare and Pharma**

**Healthcare and Data Engineering.** Advances in Internet of Things (IoT) and sensor devices have enabled integrated data processing from diverse healthcare data sources in a real-time manner [339]. In addition to existing sources (Electronic Health Record and Clinical reports), healthcare providers can use new data sources such as social media platforms, telematics, and wearable devices in order to personalize treatment plans. However, healthcare organizations face unique challenges when it comes to developing and implementing the smart health concept [11] based on using a remote cloud server with powerful computing capabilities. Besides taking into account the 3Vs (volume, velocity and variety) that raise issues related to scalability, efficiency, speed, transparency, availability, reliability, security, and others, the veracity dimension is very important because the value of health information is directly dependent on the ability to determine the quality of the data in question (accuracy, correctness, reliability). Hence, fog-enabled smart health solutions are proposed where fog nodes create a heterogeneous fog network layer and complement a portion of computation and storage of the centralized cloud server [421].

**Personalized medicine** is an approach to the practice of medicine that uses information about a patient’s unique genetic makeup and environment to customize their medical care to fit their individual requirements. Recently, epigenetics has grown in popularity as a new type of science that refers to the collection of chemical modifications to the DNA and chromatin in the nucleus of a cell, which profoundly influence the functional output of the genome. The identification of novel individual epigenetic-sensitive trajectories at the single cell level might provide additional opportunities to establish predictive, diagnostic and prognostic biomarkers as well as drug targets [386]. Based on emerging trends, patient care can be improved in many ways including using:

- modern healthcare applications that almost every smartphone possesses like Apple Health^5^, Google Health^6^ or Samsung Health^7^ are used for spotting trends and patterns;
- the data obtained by wireless body area networks, implemented with adequate permissions by the user (WBANs) can be integrated (with clinical trials, patient records, various test results and other similar data) and analysed in order to improve the effectiveness of medical institutions and to aid doctors in their decision making;
- advanced data management and processing (patient similarity, risk stratification, and treatment comparison [345]) for better prescription recommendations and optimizations of the drug supply chain, which results in cutting losses and increasing efficiency.

Over the years, the role of Artificial Intelligence in medicine has become increasingly important, for instance for image processing and diagnosis purposes. Also deep-learning neural networks have proved very useful for extracting associations between a patient’s condition and possible causes. To summarize opportunities and challenges of using innovative big data tools in healthcare, we point in Table 2 to the COVID-19 outbreak that occurred this year (Table 3).

**Table 3.** Case study: coronavirus disease 2019 (COVID-19)

Description	The outbreak of the 2019 novel coronavirus disease (COVID-19) has caused more than 5 million people to be infected and hundred of thousands of deaths. In the fight against the disease, almost all countries in the world have taken radical measures utilizing big data technologies. [485]
Key challenges	- Integration of heterogeneous data, which requires governments, businesses, and academic institutions to jointly promote the formulation of relevant policies
	- Rapid collection and aggregation of multi-source big data
	- GIS technologies for rapid visualization of epidemic information
	- Spatial tracking of confirmed cases and estimation of population flow
	- Prediction of regional transmission, spatial segmentation of the epidemic risk and prevention level
	- Balancing and management of the supply and demand of material resources

https://coronavirus-monitor.com/ (checked 22/05/2020).

**Pharma.** New trends in pharmaceutical research (such as genomic computing [370]) make the process of discovering disease patterns, early epidemic and pandemic detection and forecasting much easier. Das, Rautaray and Pandey [96] outline the general potential uses of big data in medicine like heart attack prediction, brain disease prediction, diagnosis of chronic kidney disease, analysing specific disease data, tuberculosis prediction, early hearth stage detection, HIV/AIDS prediction and some general aspects like disease outbreak and disease outcome prediction. Lee and Yoon [275] discuss some technical aspects of big data applications in medicine like missing values, the effects of high dimensionality, and bias control. Ristevski and Chen [374] mention privacy and security on the topic of big data in healthcare, while Tafti [420] offers an open source toolkit for biomedical sentence classification. Modern concepts relating to mobile health are discussed in [214] with Bayne [32] exploring big data in neonatal health care.

**Transportation and Smart Cities**

As suggested in Chap. 10.1007/978-3-030-53199-7_1, Smart Transportation is one of the key big data vertical applications besides Healthcare, Government, Energy and Utilities, Manufacturing and Natural Resources, Banking and Insurance, the Financial industry, Communications and Media, Environment and Education. The collection of related articles to this topic is possibly the largest of all applications. Zhang [483] offers a methodology for fare reduction in modern traffic congested cities, Liu [285] discusses the Internet of Vehicles, Grant-Muller [165] talks about the impacts that the data extracted from the transport domain has on other spheres, Torre-Bastida [429] talks about recent advances and challenges of modern big data applications in the transportation domain, while Imawan [211] analyses the important concept of visualization in road traffic applications. Also related, Ghofrani [154] surveys big data applications for railways, Gohar [158] discusses data-driven modelling in intelligent transportation systems, and Wang [454] attempts fuzzy control applications in this domain. Herein, we will discuss route planning applications and future challenges related to self-driving cars and user behaviour analysis.

**Route Planning Applications.** Using Global Positioning System (GPS) data, for instance, a large number of smartphone users benefit from the routing system by receiving information about the shortest or fastest route between two desired points. Some applications like Waze rely on direct user inputs in order to locate closed-off streets, speed traps etc. but at its most rudimentary level, this approach can work with just raw GPS data, calculating average travel times per street segments, and thus forming a live congestion map. Of course, such a system would be of no benefit to end users if it were not precise, but since the aggregated results that are finally presented are obtained based on many different sources, classifying this as a big data processing task, the data uncertainty is averaged out, an accurate results tend to be presented. In order to provide a quick response, geo-distributed edge devices also known as *edge servers* are used that can form an edge cloud for providing computation, storage and networking resources to facilitate big data analytics around the point of capture [91].

**Self-driving cars** rely on vast amounts of data that are constantly being provided by its users and used for training the algorithms governing the vehicle in auto-pilot mode. Holding on to the automation aspect, big data processing in the transportation domain could even be used to govern traffic light scheduling, which would have a significant impact on this sector, at least until all vehicles become autonomous and traffic lights are no longer required.

**User Behaviour Analysis.** Furthermore, the transportation domain can be optimized using adequate planning obtained from models with data originating from user behaviour analysis. Ticketing systems in countries with high population density or frequent travellers where reservations have to be made, sometimes, a few months in advance, rely on machine learning algorithms for predictions governing prices and availability. Patterns discovered from toll collecting stations and border crossings can be of huge importance when planning the duration of one’s trip and optimizing the selected route.

**Energy Production and Smart Grids**

**Energy Production.** The energy sector has been dealing with big data for decades, as tremendous amounts of data are collected from numerous sensors, which are generally attached to different plant subsystems. Recently, modern big data technologies have also been applied to plant industry such as oil and gas plants, hydro, thermal and nuclear power plants, especially in the context of improving operational performance. Thus, some of the applications of big data in the oil and gas industry [311] are analyzing seismic and micro-seismic data, improving reservoir characterization and simulation, reducing drilling time and increasing drilling safety, optimization of the performance of production pumps, improved petrochemical asset management, improved shipping and transportation, and improved occupational safety. Promising applications of big data technology in future nuclear fusion power plants are (1) data/plasma modeling in general [88], (2) real-time emergency planning [276], (3) early detection of accidents in reactors [290], etc. Related to hydro-power plants, many authors have discussed the use of IoT applications for measuring water supply (see Koo [260], Bharat [396] or Ku [418]). Zohrevand [490] talks about the application of Hidden Markov models for problem detection in systems for water supply.

**Smart Grids.** The smart grid (SG) is the next-generation power grid, which uses two-way flows of electricity and information to create a widely distributed automated energy delivery network [155]. The goal is to optimize the generation, distribution and consumption of electricity. In general, there are three main areas where data analytics have been applied:

- Ensuring smart grid stability, load forecast and prediction of energy demand for planning and managing energy network resources;
- Improving malfunction diagnosis, either on the production side (in plant facilities) or health state estimation, and identifying locations and forecasting future line outages in order to decrease the outage costs and improve system reliability;
- Profiling user behaviours to adjust individual consumption patterns and to design policies for specific users.

Smart metering equipment and sensors provide key insights into load distribution and profiles required by plant operators to sustain system stability. Predictive maintenance also plays a key role in smart grid upkeep since all segments are both critical and expensive, and any unplanned action cuts users from the electricity supply upon which almost all modern devices rely to function. Analytics methodologies or algorithms used in these cases are: 1) statistical methods; 2) signal processing methodologies; 3) supervised regression forecasting (short and long-term forecasts); 4) clustering algorithms; 4) dimensionality reduction techniques; and 5) feature selection and extraction. Tu [431] and Ghorbanian [155] present a long list of various open issues and challenges in the future for smart grids such as

- lack of comprehensive and general standard, specifically concentrated on big data management in SGs;
- interoperability of smart devices dealing with massive data used in the SGs;
- the constraint to work with approximate analytics and data uncertainty due to the increasing size of datasets and real-time necessity of processing [354];
- security and privacy issues and the balance between easier data processing and data access control for big data analytics, etc.

More insight into potential applications of big data-oriented tools and analytical technologies in the energy domain are given in Chap. 10.1007/978-3-030-53199-7_10.

**Energy Consumption and Home Automation**

An unavoidable topic when discussing big data applications, in general, is home automation. One of the challenges that the world is facing nowadays is reducing our energy consumption and improving energy efficiency. The Internet of Things, as a network of modern sensing equipment, plays a crucial role in home automation solutions that based on this data are capable of processing and providing accurate predictions, and energy saving recommendations. Home automation solutions provide optimal device scheduling to maximize comfort and minimize costs, and can even be extended from the operation aspect to planning and offering possible home adjustments or suggesting investments in renewable sources if the location being considered is deemed fit. Having smart appliances initially presented the concept of human-to-machine communication but, governed by big data processing, this concept has been further popularized with machine-to-machine communication where the human input is removed, resulting in less interference. Predictive maintenance and automatic fault detection can also be obtained from sensor data for both basic household appliances and larger mechanical systems like cars, motors, generators, etc. IoT applications require proper cloud frameworks [456]. Ge [151] presents a comprehensive survey of big data applications in the IoT sphere, Martis [300] introduce machine learning to the mix. Kumari [270] gives a survey but with the main focus on multimedia, and Kobusińska [248] talks about current trends and issues.

**Banking and Insurance**

Business intelligence tools have been used to drive profitability, reduce risk, and create competitive advantage since the 1990s. In the late 1990s, many banks and insurance companies started using machine learning techniques for categorizing and prioritizing clients, assessing the credit risk of individual clients or companies, and survival analysis, etc. As this industry generally adopts new technologies early on, thanks to advances in cognitive computing and artificial intelligence, companies can now use sophisticated algorithms to gain insights into consumer behavior. Performing inference on integrated data from internal and external sources is nowadays the key for detecting fraud and security vulnerabilities. Furthermore, novel approaches state that the applied machine learning can be supplemented with semantic knowledge, thus improving the requested predictions and classifications and enriching them with reasoning explanations that pure machine learning based deduction lacks [40]. Regarding other financial institutions, stock markets, for instance, are also a considerable use case for big data as the sheer volume and frequency of transactions slowly renders traditional processing solutions and computation methods obsolete. Finding patterns and surveilling this fast-paced process is key for proper optimization and scam prevention. Hasan [186] and Huang [204] offer concrete approaches like predicting market conditions by deep learning and applying market profile theory with Tian [427] discussing latency critical applications, Begenau [36] looking at the link between Big Data and corporate growth, and (Óskarsdóttir [492] placing an emphasis on data collected from social networks and mobile phones.

**Social Networks and e-Commerce**

**Social Networks.** When considering big data applications, one cannot overlook the massive impact that the development of social networks like YouTube, Facebook and Twitter has had on digital media and e-commerce. Social networks provide a source of personalized big data suitable for data mining with several hundreds of thousands of new posts being published every minute. They are also excellent platforms for implementing big data solutions whether it be for advertising, search suggestions, post querying or connection recommendations. The social network structure has also motivated researchers to pursue alike architectures in the big data domain. From the related literature, Saleh [381] addresses challenges in social networks that can be solved with big data, Persico [352] gives a performance evaluation of Lambda and Kappa architectures, and Ghani [152] classifies analytics solutions in the big data social media domain.

**e-Commerce.** With all services available to web users, the wide variety of online shopping websites also presents a continuous source of huge volumes of data that can be stored, processed, analysed and inferred to create recommendation engines with predictive analytics. As a means to increase user engagement, multi-channel and cross-channel marketing and analysis are performed to optimize product presence in the media fed to the user. It is no accident that a certain advertisement starts to show right after a user has searched for that specific product category. Examining user behaviour patterns and tendencies allows for offer categorization in the best possible way so that the right offer is presented precisely when it needs to be, thus maximizing sale conversions. Data received from big data analysis can also be used to govern product campaigns and loyalty programs. However, content recommendations (inferred from big data sources) in this domain are not only related to marketing and sales but are also used for proper display of information relating to the user. Some search engines companies have even publicly stated that their infrastructure relies on big data architecture, which is not surprising considering the amount of data that needs to be processed.

**Environment Monitoring**

Environmental monitoring involves the collection of one or more measurements that are used to assess the status of an environment. Advances in remote sensing using satellite and radar technologies have created new possibilities in oceanography, meteorology, forestry, agriculture and construction (urban planning). Environmental remote sensing can be subdivided into three major categories based on the distance between the sensor and the area being monitored [139]. The first category, satellite-based measurement systems, is primarily employed to study the Earth and its changing environment. The most valuable source of data from this category is the Landsat, a joint satellite program of the USGS and NASA, that has been observing the Earth continuously from 1972 through to the present day. More than 8 million images [207] are available via the NASA website^8^ and Google Earth Engine Data Catalog^9^. Additionally, the Earth observation mission from the EU Copernicus Programme produces 12 terabytes of daily observations (optical imagery at high spatial resolution over land and coastal waters) each day that can be freely accessed and analysed with DIAS, or Data and Information Access Services^10^.

The second major category of remote sensing encompasses aircraft-borne instruments, for instance, the light detection and ranging (LIDAR) systems that permit better monitoring of important atmospheric species such as ozone, carbon monoxide, water vapor, hydrocarbons, and nitrous oxide as well as meteorological parameters such as atmospheric density, pressure, and temperature [139].

Ground-based instruments (e.g. aerosols measurement instruments) and Wireless Sensor Networks (WSN) [397] are the third major category for outdoor monitoring technologies that create new opportunities to monitor farms and rain forests, cattle, agricultural (soil moisture), water quality, volcanic eruptions and earth-quakes, etc.

The table below points to some social-economic and natural environment applications enabled by big data, IoT and remote sensing (Table 4).

**Table 4.** Environment monitoring applications (examples)

Smart farming	Big data research in Smart Farming is still in an early development stage. Challenges foreseen are related both to technical and organizational issues. Technical challenges include the automation of the data acquisition process, the availability and quality of the data, and the semantic integration of these data from a diversity of sources (information on planting, spraying, materials, yields, in-season imagery, soil types, weather, and other practices). Although, from a business perspective, farmers are seeking ways to improve profitability and efficiency, there are challenges related to the governance (incl. data ownership, privacy, security) and business models for integration of the farms in the entire food supply chain [469]
Rainforest monitoring	The contribution of the world’s rainforests to the reduction of the impact of climate change is well-known to environment scientists, therefore projects have been started to integrate various low-cost sensors for measuring parameters such as humidity, temperature, total solar radiation (TSR), and photosynthetically active radiation (PAR) [68]
Biodiversity planning	- Machine learning and statistical algorithms have proved to be useful for the prediction of several numeric target attributes simultaneously, for instance, to help natural resource managers to assess vegetation condition and plan biodiversity conservation [249]

**Natural Disasters, Safety and Security**

The application of big data analytics techniques is specially important for the Safety and Security industry as it can extract hidden value (e.g. early warning, triggers, predictions) from security-related data, derive actionable intelligence, and propose new forms of surveillance and prevention. Additionally, the number of connected devices is expected to rapidly increase in the coming years with the use of AI-defined 5G networks [477]. **Natural Disasters.** Due to changing climatic conditions, natural disasters such as floods, landslides, droughts, earthquakes are nowadays becoming common events. These events create a substantial volume of data that needs to be processed in real time and thus avoid, for instance, suffering and/or death of the people affected. Advancements in the field of IoT, machine learning, big data, remote sensing, mobile applications can improve the effectiveness of disaster management strategies and facilitate implementation of evacuation processes. The requirements faced by ICT developers are similar to those in the other domains already discussed

- the need to integrate multimodal data (images, audio, text from social sites such as Twitter and Facebook);
- the need to syncronize the activities of many stakeholders involved in four aspects of emergency (preparedness, response, mitigation and recovery);
- the need to install measuring devices for collecting and real-time analysis in order to understand changes (e.g. in water level, ocean waves, ground motions, etc);
- the need to visualize information;
- the need to communicate with people (first responders and/or affected people and track their responses and behaviour) or to alert officials to initiate rescue measures.

The global market offers a wide range of emergency solutions (in the form of web and/or mobile solutions) with intuitive mapping, live field monitoring and multimedia data sharing, such as CommandWear^11^, TRACmate^12^, and Track24^13^. However, the Linked Data principles and data management techniques discussed in the previous chapters can, to a considerable extend, facilitate integration and monitoring; see for instance the *Intelligent fire risk monitor based on Linked Open Data* [442].

**Safety and Security of Critical Infrastructures.** Big data processing is especially important for protecting critical infrastructures like airports, railway/metro systems, and power grids. Large infrastructures are difficult to monitor due to their complex layout and the variety of entities that they may contain such as rooms and halls of different sizes, restricted areas, shops, etc. In emergency situations, various control and monitoring systems, e.g. fire protection systems, heating, ventilation and air conditioning systems, evacuation and access control systems and flight information display systems among others, can send altogether thousands of events to the control room each second [309]. By streaming these low-level events and combining them in a meaningful way, increased situation awareness can be achieved. Using big data tools, stream processing solutions, complex event processing/event-condition-action (CEP/ECA) paradigm and combining events, state and emergency management procedures, a wide range of emergency scenarios and emergency procedures can be pre-defined. Besides processing the large amount of heterogeneous data extracted from multiple sources while considering the challenges of volume, velocity and variety, what is also challenging today is

- real-time visualization and subsequent interaction with computational modules in order to improve understanding and speed-up decision making;
- development of advanced semantic analytics and Machine Learning techniques for new pattern recognition that will build upon pre-defined emergency scenarios (e.g. based on rules) and generate new early warning procedures or reliable action plans.

**Telecommunications**

Following the already mentioned impact of using smart mobile phones as data sources, the telecommunications industry must also be considered when discussing big data. The 5th generation of cellular network (5G) that is now live in 24 markets (GSMA predicts that it will account for 20% of global connections by 2025) will provide real-time data collection and analysis and open possibilities for business intelligence and artificial intelligence-based systems.

Mobile, television and internet service providers have customer retention as their core interest in order to maintain a sustainable business. Therefore, in order to prevent customer churn, behaviour patterns are analysed in order to provide predictions on customers looking to switch their provider and allow the company to act in time and offer various incentives or contract benefits in due course. Also, besides this business aspect, telecommunication companies using big data analytic solutions on data collected from mobile users can use the information generated in this way to assess problems with their network and perform optimizations, thus improving the quality of their service. Since almost all modern mobile phones rely on wireless 4G (and 5G in the years to come) networks to communicate when their users are not at home or work, all communication is passed through the data provider’s services, and in processing this data still lie many useful bits of information as only time will tell what useful applications are yet to be discovered. Papers covering this aspect include Yazti [479] and He [191] outlining mobile big data analytics, while Amin [15] talks about preventing and predicting the mentioned phenomena of customer churn, and Liu [286] talks about collecting data from mobile (phone and wearable) devices.

**Manufacturing**

**Fig. 2.**
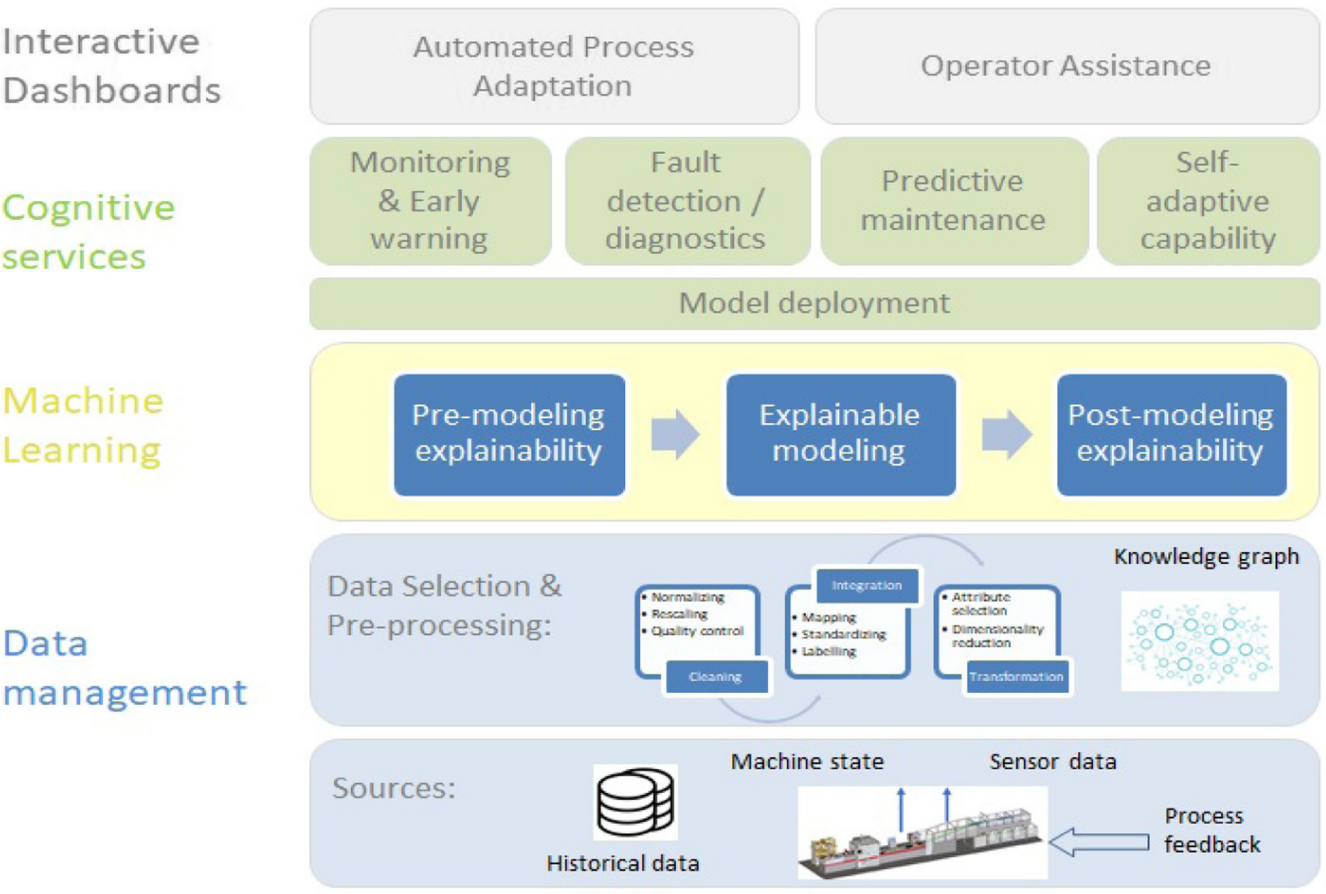
Multi-layered software architecture

Industry 4.0 is about automating processes, improving the efficiency of processes, and introducing edge computing in a distributed and intelligent manner. As discussed previously, more complex requirements are imposed in process operations while the process frequently forfeits robustness, complicating process optimization. In the Industry 4.0 era, smart manufacturing services have to operate over multiple data streams, which are usually generated by distributed sensors in almost real-time. Similarly to other industrial sectors, transforming plants into full digital production sites requires an efficient and flexible infrastructure for data integration and management connected to powerful computational systems and cognitive reasoning engines. Edge computing (distributing computing, storage, communication and control as close as possible to the mediators and objects at the edge) plays an important role in smart manufacturing. Data has to be transferred, stored, processed and transferred again back (bidirectional communications from machine to machine, machine to cloud and machine to gateway) to both users and providers in order to transmit the inferred knowledge from sensor data. In the layered infrastructure (see Fig. 2), cognitive services have a central role and their design (selection of algorithms/models) depends on the problem in place, for instance

- Kumar [268] proposes using the MapReduce framework for automatic pattern recognition based on fault diagnosis in cloud-based manufacturing. Fault diagnosis significantly contributes to reduce product testing cost and enhances manufacturing quality;
- Vater [443] discusses how new technologies, such as IoT, big data, data analytics and cloud computing, are changing production into the next generation of industry.

In the smart manufacturing ecosystem, cognitive applications make use of process data (processed on the edge) and provide high level supervisory control and support the process operators and engineers. Data analytics and AI techniques are combined with digital twins and real-life feedback from the shop floor or production facility to improve the quality of products and processes. Example areas where semantic processing and artificial intelligence can advance this sector are

- **Human-Computer Interaction.** In complex situations, operators and machines need to quickly analyze situations, communicate and cooperate with each other, coordinate emergency response efforts, and find reasonable solutions for emerging problems. In such situations, collaborative intelligence services are needed that require fewer human-driven decisions as well as easy-to-use interfaces that accelerate information-seeking and human response. Interpretability and explainability are crucial for achieving fair, accountable and transparent (FAT) machine learning, complying with the needs and standards of the business sector.
- **Dynamic process adaptation.** Many industrial processes are hard to adapt to changes (e.g. related to status and availability of all relevant production resources, or in case of anomaly detection). This affects product quality and can cause damage to equipment and production lines. Hence, a semantic framework for storing contextual information and an explainable AI approach can be used for fine-tuning of process parameters to optimize environmental resources, fast reconfiguration of machines to adapt to production change, or advance fault diagnosis and recovery.

## Conclusions

This chapter presented applications of big data approaches in different sectors. Research into real-time data analytics by addressing the volume and velocity dimension of big data is a significant area in emerging smart grid technology, for instance, where different predictive models and optimization algorithms serve to improve end-to-end performance, end-user energy efficiency and allow increasing amounts of renewable energy sources to be embedded within the distribution networks (e.g. solar photovoltaic (PV), wind power plants). Next, analytics on real-time data streams combined with GIS and weather data improves detection of significant events, enhances situational awareness and helps identify hazardous road conditions (e.g. snow), which may assist drivers and emergency responders in avoiding such conditions and allow for faster emergency vehicle routing and improved response time. Solutions that address the variety dimension, integration of heterogeneous data sources (including open and social media data) and advanced machine learning algorithms have found application in customer relation management and fraud detection (finance, insurance, telecommunication). For instance, the ability to cross-relate private information on consumer preferences and products with information from Facebook, tweets, blogs, product evaluations, and other sources opens a wide range of possibilities for organisations to understand the needs of their customers, predict their needs and demands, and optimise their use of resources. This chapter also discussed challenges that can be addressed and overcome using the semantic processing approaches and knowledge reasoning approaches discussed in this book.

## Footnotes

1 https://www.sciencedirect.com/.

2 https://link.springer.com/.

3 https://project-lambda.org/D2.1.

4 https://project-lambda.org/tools-for-experimentation.

5 https://www.apple.com/ios/health/.

6 https://health.google/.

7 https://www.samsung.com/global/galaxy/apps/samsung-health/.

8 https://landsat.gsfc.nasa.gov.

9 https://developers.google.com/earth-engine/datasets/catalog.

10 https://www.copernicus.eu/en/access-data/dias.

11 http://www.commandwear.com/features/.

12 https://play.google.com/store/apps/details?id=com.gridstone.teamactivator.

13 https://www.track24.com/smart24/.

